# Towards tuberculosis elimination in people with HIV on antiretroviral therapy: evidence from a two-decade nationwide cohort in Spain

**DOI:** 10.1186/s40249-026-01426-9

**Published:** 2026-03-06

**Authors:** Roser Navarro-Soler, Alfonso Muriel, Chiara Fanciulli, José I. Bernardino, Carmen Sáez, Javier Zamora, Borja M. Fernandez-Felix, Alejandro G. García-Ruíz de Morales, Eva Poveda, Álvaro Mena de Cea, Javier Martínez-Sanz, Santiago Moreno, Sergio Serrano-Villar

**Affiliations:** 1 Department of Infectious Diseases, Ramón y Cajal University Hospital, Ramón y Cajal Health Research Institute (IRYCIS), (CIBERINFEC), Madrid, Spain; 2 Clinical Biostatistics Unit, Ramón y Cajal University Hospital, Ramón y Cajal Health Research Institute (IRYCIS), (CIBERESP), Madrid, Spain; 3 Gregorio Marañón General University Hospital, Gregorio Marañón Health Research Institute (IiSGM), (CIBERINFEC), Madrid, Spain; 4 La Paz University Hospital, La Paz Health Research Institute (IdiPAZ), (CIBERINFEC), Madrid, Spain; 5 La Princesa University Hospital, La Princesa University Hospital Health Research Institute, Madrid, Spain; 6 Department of Clinical Postgraduate Dentistry, European University of Madrid, Madrid, Spain; 7 Galicia Sur Health Research Institute (IIS Galicia Sur), SERGAS-UVigo, Vigo, Spain; 8 A Coruña University Hospital Complex, A Coruña, Spain; 9 University of Alcalá, Madrid, Spain; 10 Life Science Campus, Antonio de Nebrija University, Madrid, Spain

**Keywords:** HIV, Tuberculosis, Antiretroviral therapy, Latent tuberculosis infection, Risk prediction, Spain, Cohort study

## Abstract

**Background:**

Tuberculosis (TB) incidence among people with HIV (PWH) has fallen in many high-income settings, yet residual risk persists, and tools to identify those still vulnerable are limited. This study aimed to evaluate TB incidence trends, identify risk factors, and develop a predictive score in PWH in Spain.

**Methods:**

We conducted a retrospective nationwide cohort study using the Spanish AIDS Research Network (CoRIS). Adults initiating antiretroviral therapy (ART) between 2004 and 2023 were followed from ART start until incident TB, death, or censoring. Incidence rates were expressed per 1000 person-years; temporal trends were evaluated across four calendar periods. Independent risk factors were identified with multivariable Cox models, and a predictive score was internally validated with bootstrapping and ROC analysis.

**Results:**

Among 16,476 PWH contributing 50,414 person-years, 61 developed TB, yielding an overall incidence of 1.21 cases/1000 person-years (95% *CI* 0.94–1.56). TB incidence rate declined by 97% between 2004–2008 and 2019–2023 (from 9.1 to 0.2 cases per 1000 person-years*; IRR* = 0.026, 95% *CI* 0.004–0.19). Injection-drug use (*HR* = 2.6, 95% *CI* 1.21–5.66), low educational attainment (*HR* = 2.8, 95% *CI* 1.05–7.51), baseline HIV-RNA viral load > 1 million copies/ml (*HR* = 1.7, 95% *CI* 1.00–2.82) and positive latent-TB infection (LTBI) screening (*HR* = 3.5, 95% *CI* 1.79–7.01) independently predicted incident TB. A four-variable model showed good discrimination (*AUC* = 0.71, 95% *CI* 0.65–0.77).

**Conclusion:**

Universal ART and comprehensive LTBI management have driven a large decrease in TB incidence in Spanish PWH, but a small subset—characterised by social vulnerability, high viraemia and positive LTBI tests—remains at elevated risk. The validated risk score offers a pragmatic tool to target preventive therapy and surveillance where most needed in low-incidence, high-care settings.

**Supplementary Information:**

The online version contains supplementary material available at 10.1186/s40249-026-01426-9.

## Background

Tuberculosis (TB) returned as the leading cause of death from an infectious agent in the world in 2023, causing almost twice as many deaths as human immunodeficiency virus (HIV)/acquired immunodeficiency syndrome (AIDS) [1]. Despite substantial progress in reducing the global burden of TB over the past decade, recent epidemiological data indicate a reversal of this downward trend, with rising incidence observed from 2021 to 2023 due to COVID-19-related disruptions in TB diagnosis and treatment. Between 2015 and 2023, global TB incidence decreased by 8.3% and deaths decreased by 23%, falling significantly below the World Health Organization (WHO) End TB Strategy milestones of 50% and 75% reduction, respectively, by 2025 [2].

HIV-TB coinfection poses a major clinical challenge, leading to more severe symptoms and faster progression of both diseases. Coinfected patients have a higher mortality likelihood than those with HIV-negative TB, even with antiretroviral treatment (ART) [3]. The risk of developing active TB increases two- to five-fold shortly after HIV-1 infection and can rise up to 20-fold in advanced stages of immunodeficiency [4]. Notably, this elevated risk persists at more than four times that of the HIV-negative general population, even after CD4⁺ T cell count (CD4) recovery with ART [3, 4].

While these challenges underscore the importance of timely diagnosis and treatment, current data reveal substantial gaps in achieving universal coverage.

In 2023, 436,805 TB cases were reported worldwide among people with HIV (PWH), representing 6.8% of all newly diagnosed TB patients with HIV test results [5, 6]. However, the WHO estimates that 662,000 PWH actually developed TB that year, meaning a substantial proportion of cases went undiagnosed. While ART coverage among reported TB-PWH was high (88%), it reached only 58% of the estimated total burden, highlighting major detection and treatment gaps particularly in resource-limited settings.

Clinically, accurately identifying PWH at high TB risk is essential, as targeted prevention is effective [7, 8]. Beyond TB preventive therapy, comprehensive strategies addressing co-infection, lifestyle risk factors, and social determinants are critical to reducing TB burden and mortality [9, 10]. While most TB-HIV research has focused on high-burden settings, low-incidence countries with universal ART access provide valuable insights into achievable TB control and residual risk factors that persist despite optimal care.

In Spain, a low TB incidence country with 8.2 cases per 100,000 population in 2023, the TB notification rate decreased by 22.5% between 2015 and 2023. Spain has an HIV prevalence of 0.3% and universal access to ART since it became available, with coverage exceeding 95% among diagnosed PWH [11]. This setting—with universal ART access and declining TB rates—provides an ideal context to examine long-term trends in TB incidence among PWH and to develop predictive models for TB risk applicable to similar low-incidence European populations. Leveraging data from the Spanish AIDS Research Network Cohort (CoRIS), we analyzed a two-decade span (2004–2023) to identify clinical and sociodemographic factors associated with TB development following ART initiation.

## Methods

### Study design and participants

We conducted a retrospective observational study using data from CoRIS, a prospective multicentre cohort comprising PWH. Between 2004 and 2023, CoRIS enrolled 20,336 ART-naive participants (defined as having no ART exposure before cohort entry) from 47 clinical centres distributed across 14 autonomous regions in Spain. Rigorous data quality controls were performed annually, with external audits conducted biennially on a 10% random sample.

We included adults (≥ 18 years) with confirmed HIV infection who initiated ART during the study period. Exclusion criteria were: (1) prevalent TB diagnosed before HIV diagnosis or within the first 6 months after ART initiation (*n* = 421), to exclude TB-associated immune reconstitution inflammatory syndrome (TB-IRIS) and unmasked pre-existing TB; (2) missing ART initiation dates; (3) patients who never initiated ART; and (4) HIV diagnosis more than four years before cohort entry, to ensure reliable and complete diagnostic information. In Spain, TB screening is systematically performed based on routine clinical assessment according to national recommendations [12]. TB diagnoses during follow-up were based on physician reporting using standardized case definitions. Ultimately, 16,476 participants met all inclusion criteria and were analyzed.

Demographic and clinical data were extracted from the CoRIS database based on previously identified predictors of TB risk in the literature, including sex, age, nationality, educational attainment (categorized as no education, primary, secondary, high school, or university), date of first positive HIV test, and route of HIV acquisition. Additionally, clinical variables including baseline CD4 counts and viral load (both measured at ART initiation), comorbidities, and latent tuberculosis infection (LTBI) screening results (tuberculin skin test [TST] or interferon-gamma release assay [IGRA] performed at cohort entry) and treatment were included due to their established association with TB susceptibility. We also included the interval between HIV diagnosis and ART initiation (ART delay) to investigate the impact of treatment delay on subsequent TB risk.

### Definitions

We defined incident TB as TB diagnosed after 6 months of ART initiation to exclude TB-IRIS, a paradoxical worsening or unmasking of pre-existing subclinical TB that occurs due to immune recovery in the first weeks to months after ART initiation [13]. Patient follow-up time was calculated in person-years, starting from the date of ART initiation until the earliest of TB diagnosis, death, or loss to follow-up.

### Outcomes

The primary outcome was the TB incidence rate among PWH receiving ART. Secondary outcomes included: cumulative probability of incident TB over time, identification of risk factors associated with incident TB development, and evaluation of temporal trends in incident TB incidence throughout the study period. Additionally, we developed and internally validated a predictive model to estimate individual risk of incident TB.

### Statistical analysis

We calculated TB incidence rates as the number of new cases per 1000 person-years of follow-up. Kaplan–Meier survival analysis was used to estimate cumulative incidence over time, and we compared groups using the log-rank test, including comparisons across calendar periods, by LTBI screening status, and by geographic origin.

We used multivariable Cox proportional hazards regression to identify independent predictors of incident TB, during the entire follow-up period, reporting results as hazard ratios (*HR*) with 95% confidence intervals (95% *CI*). Given the number of observed TB events (*n* = 61), we limited variable selection to approximately six predictors, considering their statistical significance (*P* < 0.05) and clinical relevance established in prior literature [3, 7, 14]. The final model was optimized using a backward stepwise selection approach, retaining only those variables significantly contributing to the model. Subsequently, we developed a logistic regression model for clinical prediction of incident TB using the significant predictors identified in the Cox analysis. This binary classification model was designed to estimate the probability of developing TB during the follow-up period for practical clinical application and was presented as a nomogram to facilitate risk assessment in clinical practice.

The assumption of proportional hazards was evaluated using Schoenfeld residuals. Additionally, Cox-Snell and Martingale residuals were examined to assess overall model fit. Temporal trends in TB incidence were analyzed across four 5-year intervals (2004–2008, 2009–2013, 2014–2018, 2019–2023) to capture major shifts in ART guidelines and LTBI screening implementation, and incidence rate ratios (*IRR*) and percentage changes between these intervals were calculated. Missing data for predictor variables were explicitly reported and included as separate categories in the multivariable analyses to maintain the completeness and representativeness of the cohort. Participants lost to follow-up were censored at the date of last available clinical information to accurately reflect person-time at risk.

We internally validated the predictive model using tenfold cross-validation, bootstrapping (1000 replications), and calibration assessed by the Hosmer–Lemeshow test. We evaluated model discrimination using Receiver Operating Characteristic (ROC) curves, calculating the area under the curve (*AUC*) and its 95% *CI*. Model comparison was performed using ROC curve comparison tests. Optimal cut-off points were determined by balancing sensitivity, specificity, positive predictive value, and negative predictive value. Finally, we evaluated model performance using Harrell's concordance statistic. All analyses were performed using Stata version 18.1 (StataCorp, College Station, Texas, USA).

### Ethical statement

The study protocol was approved by the Institutional Review Board of the Carlos III Health Institute, Madrid, and by the Ethics Committee of the University Hospital Ramón y Cajal (approval number 275/24). All participants included in the CoRIS cohort provided informed consent.

## Results

We evaluated a total of 16,476 PWH on ART from the CoRIS cohort between 2004 and 2023, contributing to 50,414 persons-years, of whom 61 developed incident TB. The cohort was predominantly male, with males accounting for 86% of PWH without TB and 77% of PWH with incident TB (*P* = 0.049). Age distribution significantly differed between the two groups (*P* = 0.013); notably, 51% of TB patients were aged ≥ 50 years compared to 33% among non-TB patients. Significant differences were also observed in HIV transmission routes (*P* < 0.001): while men who have sex with men (MSM) was the predominant route among non-TB patients (65%), heterosexual transmission was more prevalent among TB patients (48% vs. 28%). Immunological parameters at baseline showed no significant differences across groups; approximately 46.9% of non-TB participants and 47.5% of TB patients presented CD4 counts below 200 cells/μl (*P* = 0.51). TB patients showed a trend towards higher baseline viral loads, with 54% having > 1,000,000 copies/ml compared to 44% in non-TB patients (*P* = 0.17). Regarding LTBI screening, significant differences were observed across groups (*P* < 0.001): among TB patients, 48% had positive screening results compared to only 5% in non-TB patients (Table 1).

**Table 1:** Baseline characteristics by incident tuberculosis status

TB-PWH incident	Number (16415)	Yes (61)	*P*-value
Gender			
Male	14095 (85.9%)	47 (77.0%)	0.049
Female	2320 (14.3%)	14 (23.0%)	
Age			
18–30	1491 (9.1%)	4 (6.6%)	0.013
30–50	9494 (57.8%)	26 (42.6%)	
≥ 50	5427 (33.1%)	31 (50.8%)	
Mode of transmission			
MSM	10613 (64.7%)	23 (37.3%)	< 0.001
UDI	593 (3.6%)	8 (13.1%)	
HTS	4544 (27.7%)	29 (47.5%)	
Other/Unknown	665 (4.1%)	1 (1.6%)	
Educational level			
No education	412 (2.5%)	2 (3.3%)	0.034
Primary	1597 (9.7%)	8 (13.1%)	
Secondary	2423 (14.8%)	16 (26.2%)	
High school	4742 (28.9%)	18 (29.5%)	
University	4314 (26.3%)	6 (9.8%)	
Unknown	2927 (17.8%)	11 (18.1%)	
Baseline CD4			
CD4 < 200	7698 (46.9%)	29 (47.5%)	0.51
CD4 200–349	1240 (7.6%)	7 (11.5%)	
CD4 350–499	1793 (10.9%)	8 (13.1%)	
CD4 ≥ 500	5684 (34.6%)	17 (27.9%)	
Baseline RNA			
Unknown	292 (1.8%)	2 (3.3%)	0.17
RNA < 100000	8852 (53.9%)	26 (42.6%)	
RNA > 1000000	5136 (44.3%)	33 (54.1%)	
Smoking status			
Smoker	5136 (31.3%)	20 (32.8%)	0.14
Former smoker	1465 (8.9%)	6 (9.8%)	
Non-smoker	5328 (32.5%)	12 (19.7%)	
Unknown	4486 (27.3%)	23 (37.3%)	
LTBI screening			
Screening −	8766 (53.4%)	29 (47.5%)	< 0.001
Screening +	825 (5.3%)	12 (19.7%)	
Screening ND	6365 (41.2%)	20 (32.8%)	
Delay in ART initiation (days)			
ART delay ≤ 30	5332 (32.5%)	16 (26.2%)	0.63
ART delay 31–90	4047 (24.7%)	15 (24.6%)	
ART delay 91–180	1754 (10.7%)	6 (9.8%)	
ART delay > 180	5282 (32.2%)	24 (39.3%)	
Alcohol			
No	5426 (33.1%)	17 (27.9%)	0.39
Yes	4073 (24.8%)	13 (21.3%)	
Unknown	6916 (42.1%)	31 (50.8%)	
HCV			
Negative	14731 (89.7%)	46 (75.4%)	< 0.001
Positive	1323 (8.1%)	17 (24.6%)	
Unknown	361 (2.2%)	0 (0.0%)	
Origin			
Spain	8843 (53.9%)	31 (50.8%)	0.39
Western Europe	2179 (13.3%)	11 (18.0%)	
Eastern Europe	307 (1.9%)	1 (1.6%)	
Sub-Saharan Africa	650 (4.0%)	5 (8.2%)	
North Africa	210 (1.3%)	0 (0.0%)	
Latin America	4031 (24.6%)	12 (19.7%)	
Others	195 (1.2%)	1 (1.6%)	

*TB-PLWH* Tuberculosis in People Living with HIV, *MSM* Men who have Sex with Men, *UDI* Injecting Drug Users, *HTS* Heterosexuals, *CD4* CD4 + T lymphocyte count (cells/μl), *RNA* HIV viral load (copies/ml), *LTBI* Latent Tuberculosis Infection, *Screening ND* LTBI Screening Not Determined, *ART* Antiretroviral Therapy, *HCV* Hepatitis C Virus

### Incidence of tuberculosis

**Fig. 1:**
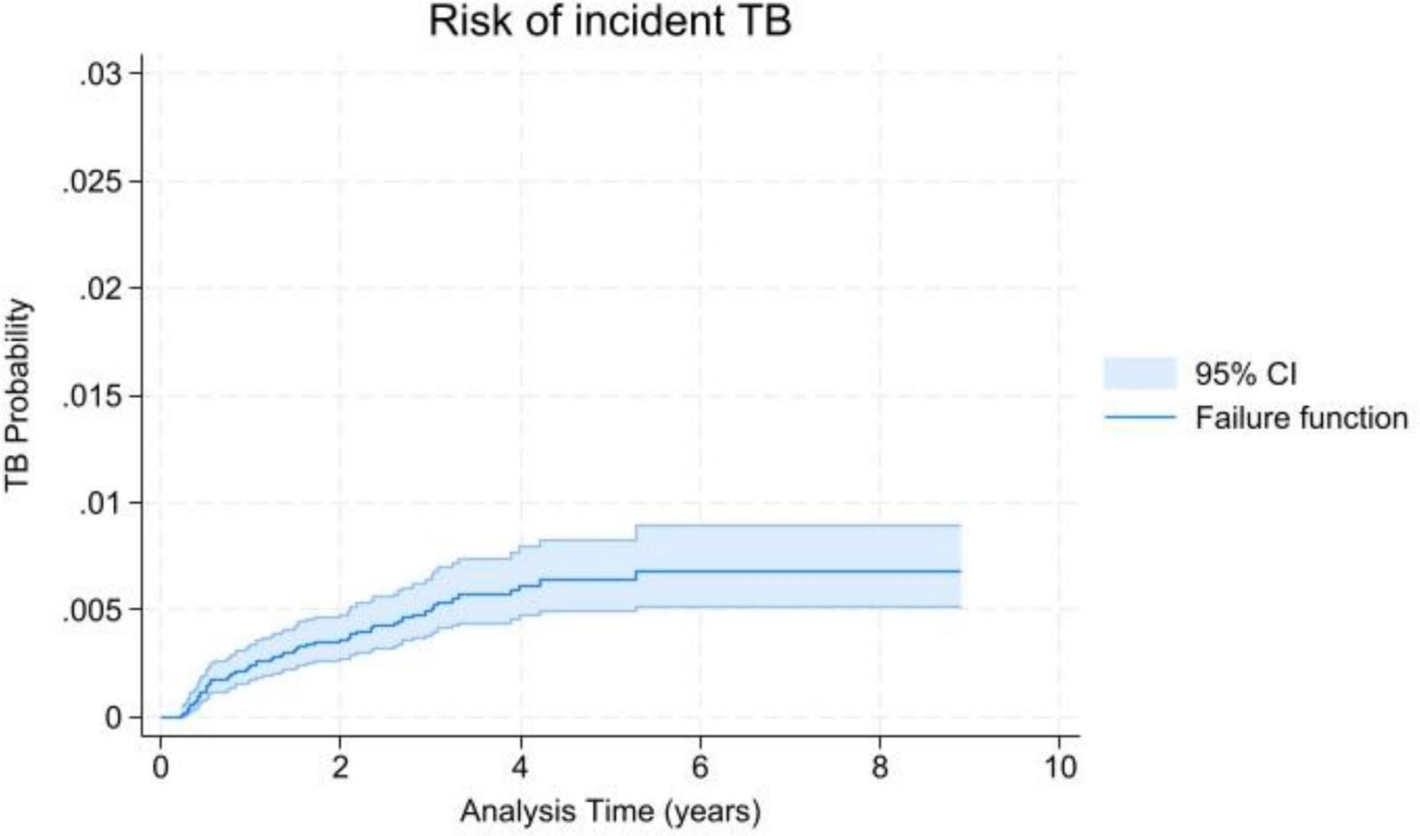
Cumulative incidence of tuberculosis: Kaplan–Meier curve showing the overall cumulative incidence of tuberculosis over 10 years of follow-up in the study cohort. Shaded area represents 95% confidence intervals

**Fig. 2:**
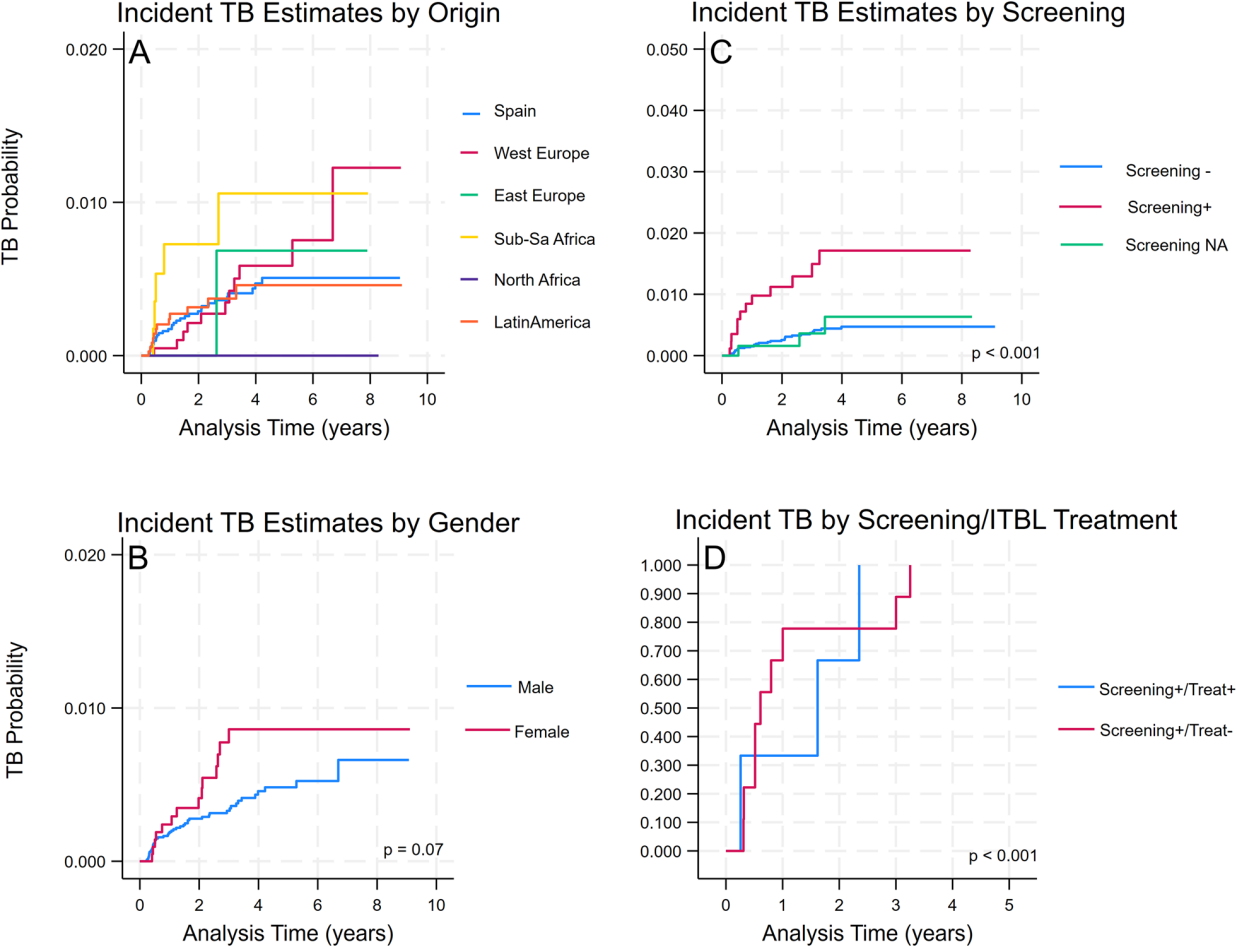
Cumulative incidence of tuberculosis by screening result, geographic origin, and gender. Cumulative incidence of tuberculosis over 5–10 years of follow-up by **A** geographic origin, **B** gender, **C** TB screening result, and **D** ITBL treatment among screening-positive patients. Higher incidence rates were observed in screening-positive individuals, patients from Sub-Saharan Africa and Latin America, and females. *P* values represent log-rank test results

The overall TB incidence following the first 6 months of ART was 1.21 cases per 1000 person-years (95% *CI* 0.94–1.56), representing a cumulative incidence of 0.37% (95% *CI* 0.28–0.48%). Kaplan–Meier analysis demonstrated a progressive increase in the cumulative probability of developing TB over follow-up: 0.19% at 1 year, 0.30% at 2 years, 0.40% at 3 years, 0.52% at 4 years, and 0.54% at 5 years (Fig. 1). We found no significant differences in TB incidence by geographic origin (*HR* range: 0.78–1.64, *P* > 0.05) or gender (*HR* = 1.48, 95% *CI* 0.78–2.79,* P* = 0.227). Participants with a positive baseline TB screening had significantly higher TB incidence, particularly among patients who did not receive LTBI treatment (Fig. 2).

### Temporal trends

**Fig. 3:**
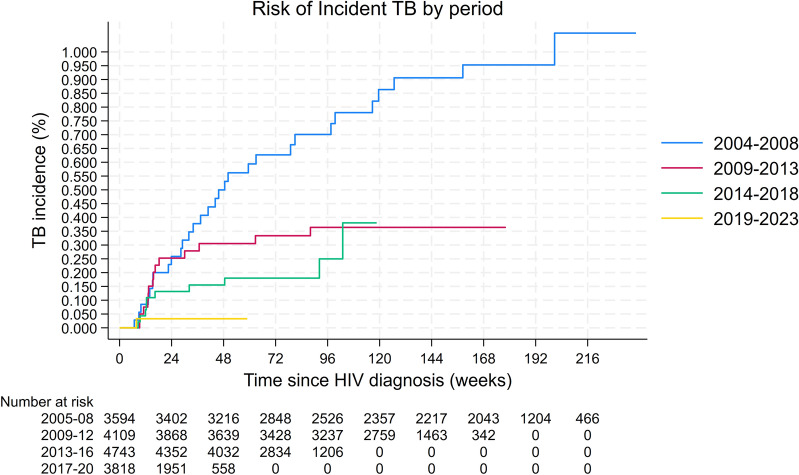
Cumulative incidence of tuberculosis among people living with HIV by period of HIV diagnosis (2004–2023). Kaplan–Meier curves show the cumulative risk of developing TB over time (in weeks) since HIV diagnosis, stratified by five calendar periods (2004–2008, 2009–2012, 2013–2016, 2017–2020, and 2020–2023). The number of patients at risk for each time period is displayed in the table below the graph, showing declining follow-up numbers over time due to events or censoring. A clear reduction in TB risk is observed across successive periods, consistent with improvements in early diagnosis and widespread antiretroviral therapy coverage

Temporal trend analysis indicated a significant reduction in TB occurrence over the study period. TB incidence decreased from 9.1 cases per 1000 person-years in 2004–2008 to 0.2 cases per 1000 person-years in 2019–2023. Compared to 2004–2008, incidence rates declined by 60% in 2009–2013 (*IRR* = 0.40, 95% *CI* 0.21–0.75,* P* = 0.004), 78% in 2014–2018 (*IRR* = 0.22, 95% *CI* 0.11–0.45,* P* < 0.001), and 97% in 2019–2023 (*IRR* = 0.026, 95% *CI* 0.004–0.19, *P* < 0.001). Linear trend analysis showed an average decline of 60% in incident TB risk with each successive period (*IRR* = 0.40, 95% *CI* 0.30–0.54, *P* < 0.001). Kaplan–Meier curves highlighted the highest cumulative incidence (~ 0.8% at 4 years) in the 2004–2008 cohort, whereas the 2019–2023 cohort exhibited the lowest risk (~ 0.1% 2 years) early in the follow-up (Fig. 3).

### Risk factors for incident tuberculosis

**Fig. 4:**
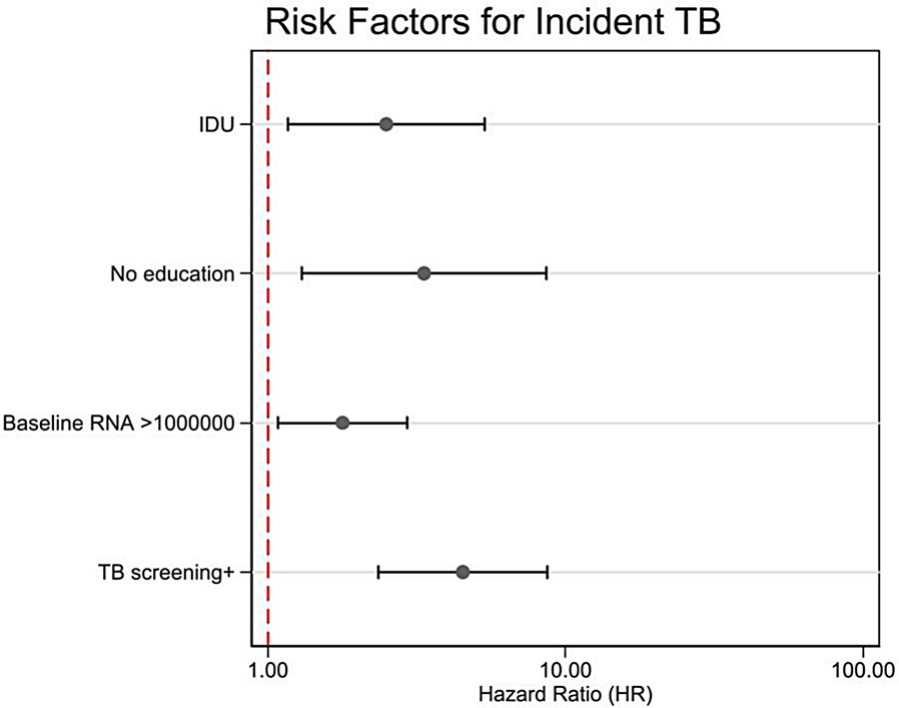
Forest plot showing adjusted hazard ratios for tuberculosis incidence in adjusted multivariable models. Forest plot showing adjusted hazard ratios (*HR*) and 95% confidence intervals from adjusted Cox regression analysis. Reference categories: non-injection drug user (IDU) transmission, university education, baseline viral load ≤ 1,000,000 copies/ml, and negative TB screening

Multivariable Cox regression identified several independent predictors of incident TB. Injection drug use (IDU) was associated with a twofold increased risk (*HR* = 2.6, 95% *CI* 1.2–5.7, *P* = 0.013). Lower educational attainment, specifically lack of tertiary education conferred significantly higher risk (*HR* = 2.7, 95% *CI* 1.02–7.25, *P* = 0.046). High baseline viral load (> 1,000,000 copies/ml) was also significantly associated with increased risk (*HR* = 1.72, 95% *CI* 1.03–2.9, *P* = 0.039). A positive baseline TB screening result was the strongest predictor, linked to over a threefold increased risk (*HR* = 3.5, 95% *CI* 1.8–7.0, *P* < 0.001). Importantly, baseline CD4 counts, smoking status did not significantly predict incident TB risk (Fig. 4).

### Predictive model for incident tuberculosis

**Fig. 5:**
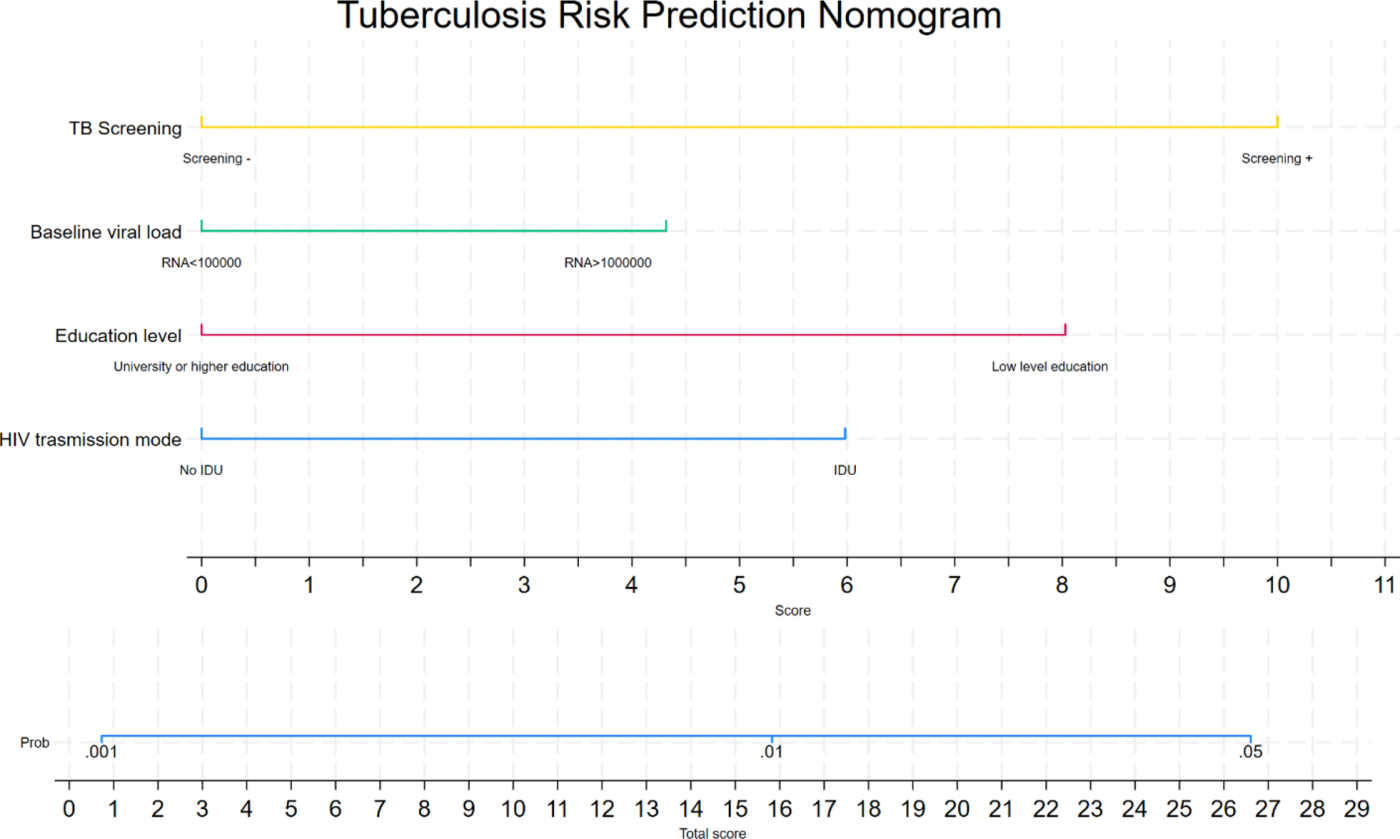
Risk prediction nomogram for incident tuberculosis. Nomogram for predicting tuberculosis incidence probability based on multivariable Cox regression. Each risk factor contributes points based on the top scale. Total points correspond to TB probability shown in the bottom scale. Higher scores indicate increased tuberculosis risk

The predictive model demonstrated good discrimination with an *AUC* = 0.71 (95% *CI* 0.65–0.77) and Harrell's C-index of 0.717 upon internal validation. The optimal cutoff value of 0.005 balanced sensitivity (58%) and specificity (73%), with a negative predictive value of 99.8%, positive likelihood ratio of 2.15, and negative likelihood ratio of 0.58, highlighting its potential clinical applicability for identifying high-risk patients. The model was presented as a nomogram for practical clinical application (Fig. 5).

Although the proportional hazards assumption was mildly violated (*P* = 0.041), residual analyses indicated minimal practical impact on model estimates (Supplementary Figure S1), and adequate model fit (Supplementary Figure S2). Model comparison analyses revealed superior performance compared to TB screening alone [*AUC* improvement from 0.59 (95% *CI* 0.52–0.66) to 0.73(95% *CI* 0.66–0.79), *P* < 0.001] (Supplementary Figure S3). This substantial 13.3-percentage-point improvement indicated that incorporating HIV transmission route, educational level, and HIV-related biomarkers significantly enhanced TB risk prediction beyond traditional screening approaches.

## Discussion

This analysis of a nationwide cohort of PWH in Spain reveals a drastic decline in TB incidence from 9.1 to 0.2 per 1000 person-years between 2004 and 2023—among the steepest drops ever reported in any HIV population. This trend outpaces previous Spanish estimates [15, 16] and most European cohorts [17], matching only the exceptional trajectory documented in Switzerland [9]. These results underscore how universal access to ART, early ART initiation regardless of immunovirological status, and robust screening for LTBI can nearly eliminate TB among PWH in low-incidence settings. Compared to other European contexts, Spain now reports markedly lower TB rates in PWH, suggesting that integrated HIV/TB care delivery can significantly alter outcomes. Given that HIV-associated TB remains a major cause of mortality globally [18]—with many cases diagnosed only post-mortem [13]—this progress is not merely epidemiological but clinically meaningful. TB leaves long-term sequelae [19, 20], reinforcing the imperative to sustain and replicate such declines.

Building on these findings, our study identifies several independent risk factors for incident TB that reflect a shift from classical immunological predictors toward social and virological determinants. IDU conferred a significantly elevated TB risk, consistent with prior findings, likely due to intersecting vulnerabilities such as homelessness, incarceration, and co-infections [21]. Similarly, lower educational attainment independently predicted TB, highlighting the influence of social determinants such as health literacy and socioeconomic status [22]. High levels of baseline viral load also emerged as a predictor of TB, reflecting ongoing immunosuppression before ART takes full effect, and possibly delayed diagnosis or missed LTBI treatment [14]. These observations align with known mechanisms—such as immune activation favouring TB reactivation [23]—and suggest that virological and social factors increasingly shape TB risk in settings with effective HIV care. Interestingly, traditional predictors like CD4 count commonly cited in high-burden or resource-limited settings [21, 22] were not significant in our cohort. This divergence may reflect the rapid immune recovery facilitated by prompt ART in Spain, rendering classical immunological markers less informative [24]. Emerging data from other low-incidence contexts [14, 25, 26] support this evolving risk landscape, indicating that TB prevention must now consider LTBI status and social determinants alongside HIV clinical markers [27–29].

To improve TB risk discrimination, we explored additional prognostic factors. While a positive LTBI screening result in our cohort remained a strong predictor of future TB, relying solely on it failed to identify at-risk individuals with negative baseline tests. Incorporating clinical, virological, and socio-demographic variables into a multivariable model significantly improved predictive accuracy compared to LTBI screening alone. These findings align with growing evidence supporting targeted over universal screening, particularly in low-incidence settings [25, 27]. WHO symptom-based screening has shown suboptimal sensitivity in PWH, leading to recommendations favoring molecular testing in hospitalized HIV-positive individuals in low-burden settings [30, 31]. The diagnostic performance of existing LTBI tools such as IGRA and TST varies widely [32], and emerging technologies—including stool-based polymerase chain reaction (PCR) and urinary biomarkers—may enhance detection among immunosuppressed patients [33, 34]. The use of predictive models like ours could help allocate resources more efficiently and personalize screening strategies, a critical need where universal testing may no longer be cost-effective [35, 36]. Nevertheless, implementation remains challenging. Studies show persistent gaps between recommendations and practice, including physicians’ limited confidence in test interpretation [37]. These issues are especially relevant in low-incidence countries, where tailoring interventions to the local epidemiological context is essential [38]. Among high-risk groups, migrants deserve particular attention, given their disproportionate burden of HIV/TB co-infection across Europe, with prevalence ranging from 1 to 76.6%—consistently higher than in native-born populations [39]. This highlights the importance of culturally sensitive, context-specific prevention approaches.

In this context, risk models can complement diagnostic tests by helping to identify which IGRA-negative individuals may still benefit from prophylaxis—or conversely, which IGRA-positive patients may be safely observed. The model’s strong negative predictive value suggests that a substantial fraction of PWH can be confidently classified as low-risk, supporting a potential shift toward less intensive monitoring in these individuals. In the setting of effective ART, this could allow for longer intervals between diagnostic assessments, thereby optimizing resource use without compromising patient safety. At the same time, focused attention on high-risk groups—such as migrants from endemic regions, individuals with histories of IDU, or those with socioeconomic vulnerability—could maximize the impact of limited preventive resources. Importantly, our model may offer clinicians a tool to navigate the persistent implementation gap in Europe, where many HIV clinics still fall short of guideline-based LTBI screening and therapy [24, 35]. Physician hesitation around test interpretation and drug toxicity may be eased by a model that prioritizes prevention in those most likely to benefit. This approach also aligns with global calls to tailor TB interventions to local epidemiology, especially in vulnerable subpopulations like migrants, among whom TB/HIV coinfection remains disproportionately high [37].

Some limitations must be acknowledged. The observational nature of this study precludes causal inference, and residual confounding may influence associations. The low number of incident TB cases limited our ability to detect weaker predictors. We also lacked granular data on LTBI testing modality (TST vs. IGRA), TB preventive therapy uptake, and treatment completion, which constrains deeper analysis of specific interventions. Moreover, while internally validated, our model needs external validation, especially in settings with different TB incidence or patient demographics. Nonetheless, the study’s strengths are considerable. It draws on a large, multicenter cohort (over 15,000 PWH from 47 sites), with high-quality standardized data and over 20 years of follow-up. This allowed precise estimation of incidence trends and multivariable modeling.

Looking ahead, TB incidence among PWH in Spain has dropped to historically low levels, demonstrating what is achievable through integrated care, early ART, and effective LTBI management. Yet our analysis reveals that TB risk persists in specific subgroups—especially those with histories of drug use, low education, and high HIV viremia at diagnosis. These factors can help identify the minority of PWH still at elevated TB risk despite effective ART. Our multivariable prediction model significantly outperformed LTBI screening alone in stratifying TB risk and offers a promising framework for targeted prevention. These findings support recalibrating TB preventive guidelines in low-incidence countries toward a more nuanced, context-sensitive approach. Further research should prospectively evaluate the implementation of such models in clinical settings and validate them across diverse populations. Ultimately, the Spanish experience—conducted in a low TB incidence setting with a universal healthcare system and among PWH receiving ART—highlights a path to achieving residual TB levels through comprehensive and equitable HIV/TB.

## Conclusions

TB incidence among PWH in Spain has fallen to historically low levels through universal ART and integrated HIV/TB care, demonstrating what can be achieved with sustained, equitable public health efforts. However, residual risk remains among socially and virologically vulnerable individuals, underscoring the need for prevention strategies that go beyond biomedical interventions. The predictive model developed in this study complements LTBI screening and provides a practical framework to optimize resource allocation and guide targeted prophylaxis in low-incidence settings. Sustaining these achievements will require continued attention to social determinants, early ART initiation, and evidence-based implementation adapted to local epidemiology.

## Supplementary Information

Supplementary material 1: Figure S1. Proportional hazards assessment. Panel A Log-log survival curves. Panel B: Schoenfeld residuals analysis. Proportional hazards assessment. Panel A shows log-log survival curves indicating statistical violation for TB screening. Panel B demonstrates flat Schoenfeld residual trends confirming minimal practical impact.

Supplementary material 2: Figure S2. Model goodness-of-fit. Panel A: Martingale residuals. Panel B: Cox-Snell residuals. Model validation showing appropriate fit and specification.

Supplementary material 3: Figure S3. Comparison of ROC curves for tuberculosis prediction models. ROC curves comparing the discriminative performance of the base model (*AUC* = 0.59, 95% *CI*: 0.52–0.66) versus the extended model (*AUC *= 0.73, 95% *CI*: 0.66–0.79) for incident tuberculosis prediction in HIV-positive patients. The reference line represents random classification (*AUC* = 0.50).

Supplementary material 4.

## Data Availability

The datasets generated and/or analyzed during the current study are available from the corresponding author on reasonable request.

## Abbreviations

AIDS: Acquired immunodeficiency syndrome
ART: Antiretroviral therapy
AUC: Area under the curve
CD4: CD4⁺ T-cell count
CI: Confidence interval
CoRIS: Spanish AIDS Research Network Cohort
HIV: Human immunodeficiency virus
HR: Hazard ratio
IDU: Injection drug use
IGRA: Interferon-gamma release assay
IRR: Incidence rate ratio
LTBI: Latent tuberculosis infection
MSM: Men who have sex with men
PCR: Polymerase chain reaction
PWH: People with HIV
ROC: Receiver operating characteristic
TB: Tuberculosis
TB-IRIS: TB-associated immune reconstitution inflammatory syndrome
TST: Tuberculin skin test
WHO: World Health Organization
