# Supplementary figures and images for: Towards tuberculosis elimination in people with HIV on antiretroviral therapy: evidence from a two-decade nationwide cohort in Spain
Source: Infect Dis Poverty. 2026 Mar 6;15:30. doi: 10.1186/s40249-026-01426-9 (PMC12964894; doi:10.1186/s40249-026-01426-9)

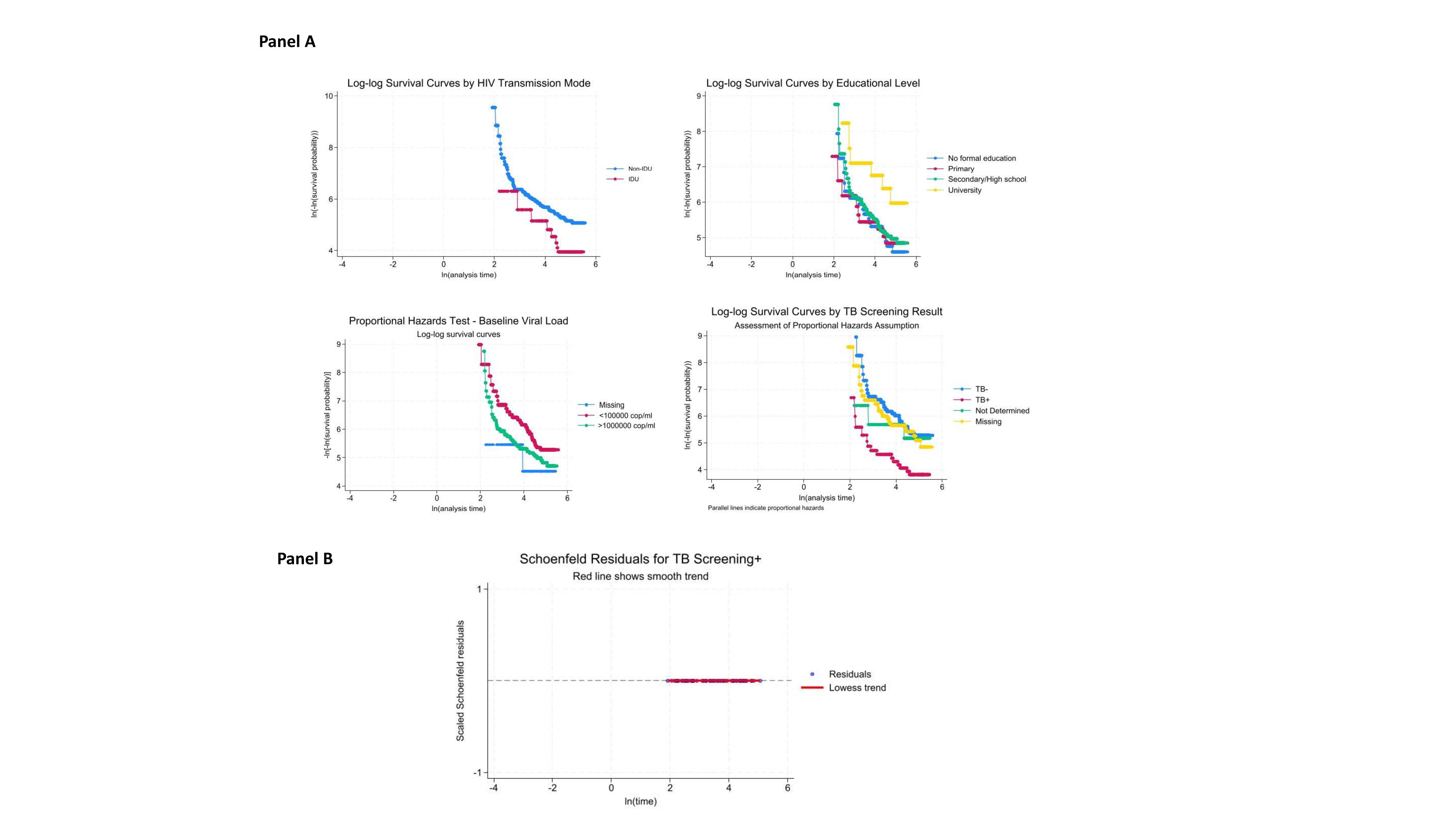

Supplement: Supplementary file 1 — Supplementary material 1: Figure S1. Proportional hazards assessment. Panel A Log-log survival curves. Panel B: Schoenfeld residuals analysis. Proportional hazards assessment. Panel A shows log-log survival curves indicating statistical violation for TB screening. Panel B demonstrates flat Schoenfeld residual trends confirming minimal practical impact. [file 40249_2026_1426_MOESM1_ESM.jpg]

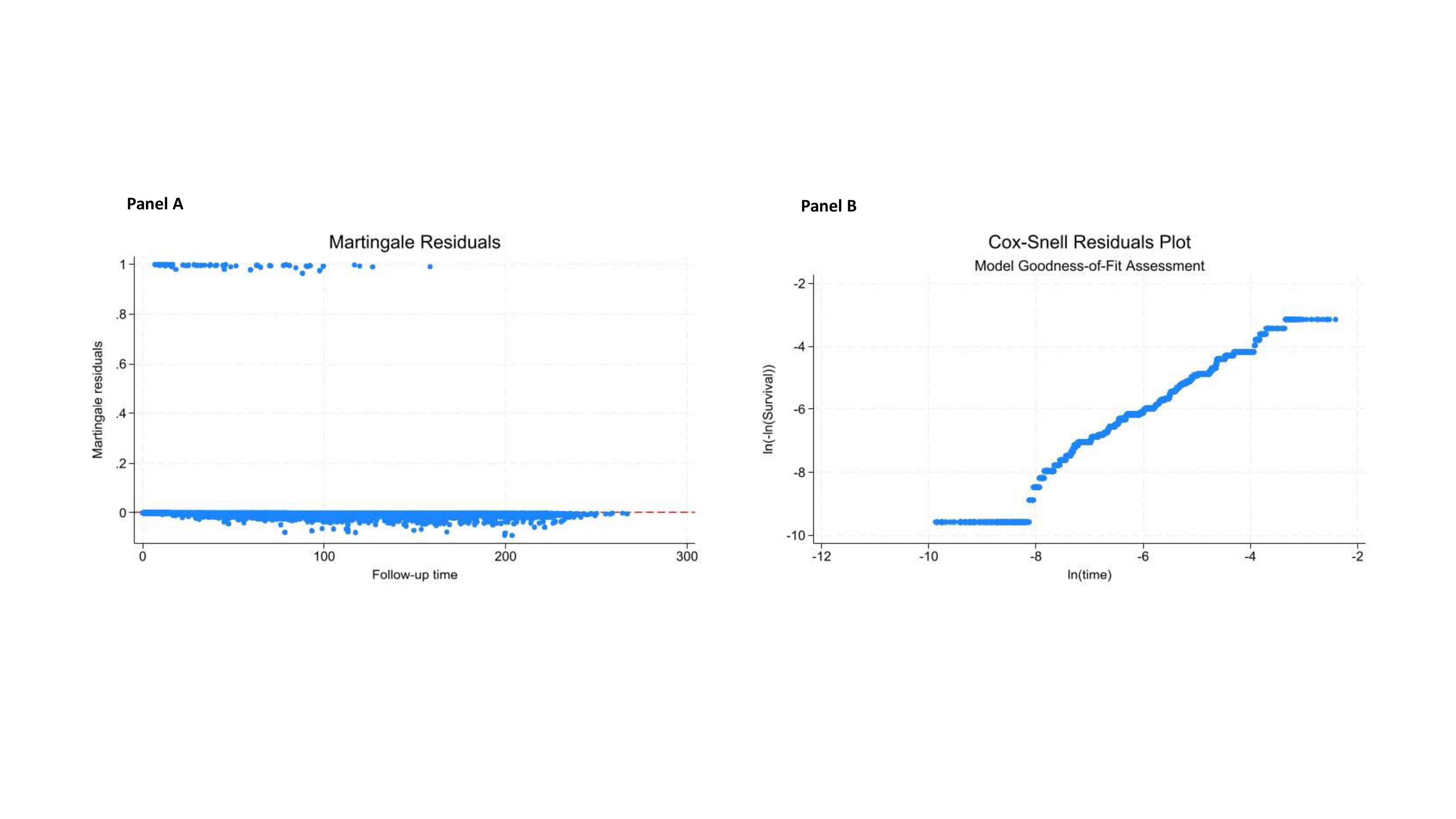

Supplement: Supplementary file 2 — Supplementary material 2: Figure S2. Model goodness-of-fit. Panel A: Martingale residuals. Panel B: Cox-Snell residuals. Model validation showing appropriate fit and specification. [file 40249_2026_1426_MOESM2_ESM.jpg]

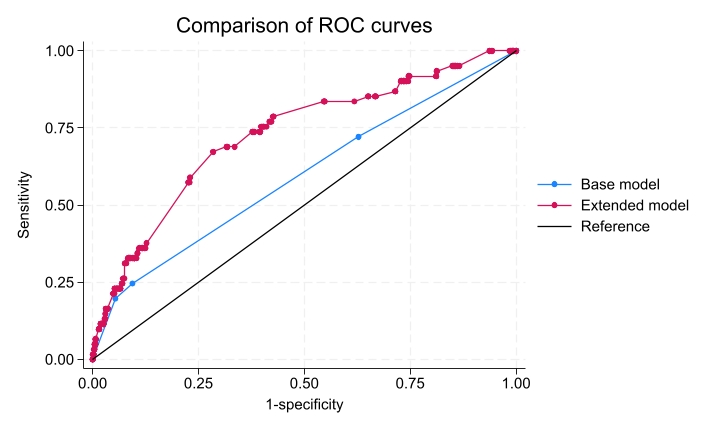

Supplement: Supplementary file 3 — Supplementary material 3: Figure S3. Comparison of ROC curves for tuberculosis prediction models. ROC curves comparing the discriminative performance of the base model (AUC = 0.59, 95% CI: 0.52–0.66) versus the extended model (AUC = 0.73, 95% CI: 0.66–0.79) for incident tuberculosis prediction in HIV-positive patients. The reference line represents random classification (AUC = 0.50). [file 40249_2026_1426_MOESM3_ESM.jpg]
