# Supplementary material for: Towards tuberculosis elimination in people with HIV on antiretroviral therapy: evidence from a two-decade nationwide cohort in Spain
Source: Infect Dis Poverty. 2026 Mar 6;15:30. doi: 10.1186/s40249-026-01426-9 (PMC12964894; doi:10.1186/s40249-026-01426-9)
Supplement: Supplementary file 4 — Supplementary material 4. [file 40249_2026_1426_MOESM4_ESM.docx]

***CoRIS Executive committee:***

Santiago Moreno, Inma Jarrín, David Dalmau, M Luisa Navarro, M Isabel González, Federico Garcia, Eva Poveda, Jose Antonio Iribarren, Félix Gutiérrez, Rafael Rubio, Francesc Vidal, Juan Berenguer, Juan González, M Ángeles Muñoz-Fernández.

***Centres and investigators involved in CoRIS cohort are listed below:***

**CoRIS Coordination Unit**

Inmaculada Jarrín, Cristina Moreno, Marta Rava, Rebeca Izquierdo, Cristina Marco, Teresa Gómez-García.

**BioBanK HIV Hospital General Universitario Gregorio Marañón**

Mª Ángeles Muñoz-Fernández, Roxana Juárez.

**Hospital General Universitario Dr Balmis (Alicante)**

Joaquín Portilla, Irene Portilla, Esperanza Merino, Gema García, Iván Agea, José Sánchez-Payá, Juan Carlos Rodríguez, Livia Giner, Sergio Reus, Vicente Boix, Diego Torrus, Verónica Pérez, Julia Portilla, Héctor Pinargote.

**Hospital Universitario de Canarias (San Cristóbal de la Laguna)**

María Remedios Alemán, Ana López Lirola, Dácil García, Felicitas Díaz-Flores, M Mar Alonso, Ricardo Pelazas, María Inmaculada Hernández, Lucia Romero, Abraham Bethencourt, Daniel Rodríguez.

**Hospital Universitario Central de Asturias (Oviedo)**

Víctor Asensi, Rebeca Cabo Magadan, Lorena Fernández, Javier Díaz-Arias

**Hospital Universitario 12 de Octubre (Madrid)**

Federico Pulido, Rafael Rubio, Otilia Bisbal, M Asunción Hernando, David Rial, María de Lagarde, Adriana Pinto, Laura Bermejo, Mireia Santacreu, Roser Navarro, Juan Martín Torres.

**Servicio de Enfermedades Infecciosas. Hospital Universitario Donostia. Instituto de Investigación BioDonostia (Donostia-San Sebastián)**

José Antonio Iribarren, M José Aramburu, Xabier Camino, Miguel Ángel Goenaga, M Jesús Bustinduy, Harkaitz Azkune, Maialen Ibarguren, Xabier Kortajarena, Ignacio Álvarez-Rodriguez, Leire Gil, Francisco Carmona-Torre, Ana Bayona Carlos, Maialen Lekuona Sanz, Claudia Nevado Pavón.

**Hospital General Universitario De Elche (Elche)**

Félix Gutiérrez, Catalina Robledano, Mar Masiá, Sergio Padilla, Rafael Pascual, Marta Fernández, Antonio Galiana, José Alberto García, Xavier Barber, Javier García Abellán, Guillermo Telenti, Ángela Botella, Paula Mascarell, Mar Carvajal, Lidia García-Sánchez, Nuria Ena, Leandro López, Jennifer Vallejo, Nieves Gonzalo-Jiménez, Montserrat Ruiz, Christian Ledesma, Santiago López, María Espinosa, Ana Quiles, María Andreo, María del Mar Alcalde, José García, Rosario Hernández, José Carlos Escribano, Marouane Menchi, María del Mar García Navarro.

**Hospital General Universitario Gregorio Marañón (Madrid)**

Juan Carlos López Bernaldo de Quirós, Isabel Gutiérrez, Juan Berenguer, Margarita Ramírez, Paloma Gijón, Teresa Aldamiz-Echevarría, Francisco Tejerina, Cristina Diez, Leire Pérez, Chiara Fanciulli, Saray Corral.

**Hospital Universitari de Tarragona Joan XXIII (Tarragona)**

Joaquín Peraire, Anna Rull, Anna Martí, Consuelo Viladés, Beatriz Villar, Lluïsa Guillem, Silvia Chafino, Marina Flores.

**Hospital Universitario y Politécnico de La Fe (Valencia)**

Marta Montero-Alonso, María Tasias, Eva Calabuig, Miguel Salavert, Juan Fernández, Rosa Blanes, Jennifer Sánchez.

**Hospital Universitario La Paz/IdiPAZ (Madrid)**

Juan González-García, Ana Delgado-Hierro, José Ramón Arribas, Víctor Arribas, José Ignacio Bernardino, Carmen Busca, Joanna Cano-Smith, Julen Cadiñanos, Juan Miguel Castro, Luis Escosa, Iker Falces, Pedro Herranz, Víctor Hontañón, Alicia González-Baeza, M Luz Martín-Carbonero, Mario Mayoral, Rafael Micán, Rosa de Miguel, Rocío Montejano, Mª Luisa Montes, Luis Ramos-Ruperto, Berta Rodés, Talía Sainz, Elena Sendagorta, Eulalia Valencia, M del Mar Arcos, Alejandro de Gea Grela, Carlos Oñoro López.

**Hospital Universitari Mutua Terrassa (Terrassa)**

David Dalmau, Marina Martinez, Angels Jaén, Mireia Cairó, Javier Martinez-Lacasa, Roser Font, Laura Gisbert.

**Hospital Universitario de La Princesa (Madrid)**

Ignacio de los Santos, Alejandro de los Santos, Lucio García-Fraile, Enrique Martín, Ildefonso Sánchez-Cerrillo, Marta Calvet, Ana Barrios, Azucena Bautista, Carmen Sáez, Marianela Ciudad, Ángela Gutiérrez, María Aguilera García, Violeta Sampériz Rubio.

**Hospital Universitario Ramón y Cajal (Madrid)**

Santiago Moreno, Santos del Campo, José Luis Casado, Fernando Dronda, Ana Moreno, M Jesús Pérez, Sergio Serrano-Villar, Mª Jesús Vivancos, Javier Martínez-Sanz, Alejandro Vallejo, Matilde Sánchez, José Antonio Pérez-Molina, José Manuel Hermida, Erick De La Torre Tarazona, Elena Moreno, Laura Martín Pedraza, Claudio Díaz García, Jorge Díaz, Alejandro García, Raquel Ron.

**Hospital General Universitario Reina Sofía (Murcia)**

Enrique Bernal, Antonia Alcaraz, Joaquín Bravo, Ángeles Muñoz, Cristina Tomás, Eva Oliver, Eva García, Román González, Elena Guijarro, Rodrigo Martínez, María Dolores Hernández.

**Hospital Universitario Clínico San Cecilio (Granada)**

Federico García, Clara Martínez, Leopoldo Muñoz Medina, Marta Álvarez, Natalia Chueca, David Vinuesa, Adolfo de Salazar, Ana Fuentes, Emilio Guirao, Andrés Ruiz-Sancho, Francisco Anguita, Naya Faro, Lucia Chaves, Marta Illescas, Paloma Muñoz, Lucía Pérez.

**Centro Sanitario Sandoval (Madrid)**

Jorge Del Romero, Montserrat Raposo, Teresa Puerta, Mar Vera, Juan Ballesteros, Begoña Baza, Eva Orviz, Manuel Sanchez Robledo, Laura Dans Villán, Ruben Linares Navarro. Ines Armenteros Yeguas.

**Hospital Universitario Son Espases (Palma de Mallorca)**

Melchor Riera, María Peñaranda, M Angels Ribas, Antoni A. Campins, Mercedes Garcia-Gasalla, Francisco J Fanjul, Javier Murillas, Luisa Martin-Pena, Francisca Artigues, Sophia Pinecki.

**Hospital Universitario Virgen de la Victoria (Málaga)**

Jesús Santos, María López-Jódar, Cristina Gómez-Ayerbe, Isabel Viciana, Rosario Palacios.

**Hospital Universitario Virgen del Rocío (Sevilla)**

Luis Fernando López-Cortés, Nuria Espinosa, Cristina Roca, Silvia Llaves, Marta Herreros, César Sotomayor.

**Hospital Universitario de Bellvitge (Hospitalet de Llobregat)**

Juan Manuel Tiraboschi, Arkaitz Imaz, María Saumoy, Analuz Fernandez, Jaime Vega Costa, Daniel Medina Gamito.

**Hospital Costa del Sol (Marbella)**

Julián Olalla, Javier Pérez, Alfonso del Arco, Javier de la Torre, José Luis Prada.

**Hospital General Universitario Santa Lucía (Cartagena)**

Onofre Juan Martínez, Lorena Martinez, Francisco Jesús Vera, Josefina García, Begoña Alcaraz, Sergio Guillén Martínez, Patricia Carles García.

**Complejo Hospitalario Universitario a Coruña (CHUAC) (A Coruña)**

Álvaro Mena, Berta Pernas, Pilar Vázquez, Soledad López, Brais Castelo.

**Hospital Universitario Virgen de la Arrixaca (El Palmar)**

Carlos Galera, Marian Fernández, Helena Albendin, Antonia Castillo, Asunción Iborra, Antonio Moreno, M Angustias Merlos, Almudena Ortuño.

**Hospital Universitario Infanta Sofía (San Sebastián de los Reyes)**

Inés Suarez-García, Eduardo Malmierca, Patricia González-Ruano, M Pilar Ruiz, Luz Balsalobre, Ángela Somodevilla, Rebeca Fuerte Martínez.

**Hospital Clínico San Carlos (Madrid)**

Vicente Estrada, Noemí Cabello, María José Núñez, Juncal Pérez-Somarriba, Reynaldo Homen, Ana Muñoz, Julia Barrado, Maravillas Carralon, Nieves Sanz, Susana Olmedo.

**Hospital Universitario Príncipe de Asturias (Alcalá de Henares)**

José Sanz, Cristina Hernández, María Novella.

**Hospital Clínico Universitario de Valencia (Valencia)**

María José Galindo, Sandra Pérez Gómez, Ana Ferrer.

**Hospital Reina Sofía (Córdoba)**

Antonio Rivero Román, Antonio Rivero Juárez, Pedro López, Mario Frias, Ángela Camacho, Ignacio Pérez, Diana Corona, Javier Manuel Caballero, Laura Ruiz Torres, Ángela Carrasco, Marina Gallo Marín, María Casares, Lucía Rios Muñoz

**Hospital Universitario Severo Ochoa (Leganés)**

Rafael Rodríguez-Rosado Martinez-Echevarría, Rafael Torres.

**Hospital Universitario Virgen de Valme (Sevilla)**

Juan Macías Sánchez, Pilar Rincón, Luis Miguel Real, Anaïs Corma, Jésica Martín.

**Hospital Álvaro Cunqueiro (Vigo)**

Eva Poveda, Alexandre Pérez, Luis Morano, Celia Miralles, Antonio Ocampo, Jacobo Alonso, Inés Martínez, Aida López.
